# Promiscuous drugs compared to selective drugs (promiscuity can be a virtue)

**DOI:** 10.1186/1472-6904-5-3

**Published:** 2005-04-26

**Authors:** Simon K Mencher, Long G Wang

**Affiliations:** 1 The NYU Cancer Institute, New York University School of Medicine, Manhattan VA Hospital, 18003 W, 423 East 23^rd ^Street, NY, NY 10010, USA

## Abstract

**Background:**

The word selectivity describes a drug's ability to affect a particular cell population in preference to others. As part of the current state of art in the search for new therapeutic agents, the property of selectivity is a mode of action thought to have a high degree of desirability. Consequently there is a growing activity in this area of research.

Selectivity is generally a worthy property in a drug because a drug having high selectivity may have a dramatic effect when there is a single agent that can be targeted against the appropriate molecular-driver involved in the pathogenesis of a disease. An example is chronic myeloid leukemia (CML). CML has a specific chromosomal abnormality, the Philadelphia chromosome, that results in a single gene that produces an abnormal protein

**Discussion:**

There is a burgeoning understanding of the cellular mechanisms that control the etiology and pathogeneses of diseases. This understanding both enables and motivates the development of drugs that induce a specific action in a selected cell population; i.e., a targeted treatment. Consequently, drugs that can target distinct molecular targets involved in pathologic/pathogenetic processes, or signal-transduction pathways, are being developed.

However, in most cases, diseases involve multiple abnormalities. A disease may be associated with more than one dysfunctional protein and these may be out-of-balance with each other. Likewise a drug might strongly target a protein that shares a similar active domain with other proteins. A drug may also target pleiotropic cytokines, or other proteins that have multi-physiological functions. In this way multiple normal cellular pathways can be simultaneously influenced. Long term experience with drugs supposedly designed for only a single target, but which unavoidably involve other functional effects, is uncovering the fact that molecular targeting is not medically flawless.

**Summary:**

We contend that an ideal drug may be one whose efficacy is based not on the inhibition of a single target, but rather on the rebalancing of the several proteins or events, that contribute to the etiology, pathogeneses, and progression of diseases, i.e., in effect a promiscuous drug. Ideally, if this could be done at minimum drug concentration, side effects could be minimized. Corollaries to this argument are that the growing fervor for researching truly selective drugs may be imprudent when considering the totality of responses; and that the expensive screening techniques used to discover these, may be both medically and financially inefficient.

## Background

The words selectivity, specificity, and sensitivity (derived from Latin seligere, specificus, sensitivus), can be confusing terms as they are often used synonymously in the medical literature. However, they should not be used interchangeably as each represents a different phenomenon. For the sake of consistency and clarity, this paper will use the terms as defined below:

Selectivity will be used to describe the ability of a drug to affect a particular population, i.e., gene, protein, signaling pathway, or cell, in preference to others. For example a selective drug would have the ability to discriminate between, and so affect only one cell population, and thereby produce an event.

Specificity, a term most often confused with selectivity, will be used to describe the capacity of a drug to cause a particular action in a population. For example, a drug of absolute specificity of action might decrease or increase, a specific function of a given gene or protein or cell type, but it must do either, not both.

Sensitivity will be used to describe the capacity of a population, to respond to a drug's ability, to stimulate that entity at a specified dose. The smaller the dose required producing an effect, the more sensitive is the responding system. (The word used to describe this activity in the drug which is the cause of the population sensitivity, is potency).

It can be seen therefore, that a drug's activity may involve all the above attributes-it may be selective to one cell population, and also be specific to one kind of action on that cell population, and the population in turn, may be sensitive to the drug's influence at a lower dose than would other responding systems.

As part of the current state of art in the search for new therapeutic agents, the property of selectivity is a mode of action thought to have a high degree of desirability and there is a great deal of activity in this area of research. It is the growing understanding of the cellular mechanisms that control etiology of diseases together with modern technology that enables and motivates the development of targeted therapies. This search for selective drugs has led to the development of high-throughput, virtual screening, and rational drug design techniques that are widely used to discover leads for drug candidates. Successes have lead to small molecular drugs that can target specific proteins involved in signal-transduction pathways leading to pathogenesis. Indeed, a drug having high selectivity may have a dramatic effect when there is a single agent that can be targeted against the appropriate molecular-driver involved in the pathogenesis of a disease. An example is chronic myeloid leukemia that has a specific chromosomal abnormality called the Philadelphia chromosome that results in a gene that produces an abnormal protein. However a protein being targeted may share a similar active domain with other proteins having normal physiologic functions; hence this would cause undesirable side effects. Long term experience with drugs supposedly designed for only a single target, but which unavoidably involve other functional effects, is uncovering the fact that molecular targeting is not medically flawless. The recent discovery related to Cox -2 inhibition is the most striking example [2].

## Discussion

Selectivity is generally a worthy property in a drug, e.g., it is desirable to have a chemotherapy drug to affect prostate cancer cells and not affect nearby healthy prostate cells and other normal tissues. Similarly an anti-bacterial for germs or parasites should be suitably potent so that it can be used in a small dose sufficient to kill the infectious agents but not to affect host cells. Sometimes, however, selectivity is undesirable as in a case where a drug strongly targets pleiotropic cytokines or other proteins that have multifunctional effects and so can influence multiple cellular pathways simultaneously. Important examples are TNF-α and cyclic AMP, both of which have a variety of effects in a cell, pathogenetically and physiologically. TNF-α has a dual nature in cell death and cAMP acts to control a protein kinase that in turn affects activities of a variety of cellular proteins [3-5].

The strategy for selective drug development is based on the abundant progress made in the last decade in human genomic and proteomic projects. For example, a major related effort is structure-based drug design. Here the three-dimensional structure of a drug target interacting with small molecules is used to guide drug discovery. Structure-based design enables a researcher to "see" exactly how a molecule interacts with its target protein and so bind a selective agent to the target.

Specifically, cancer treatment is rapidly evolving from systemic, non-specific, high-dose chemotherapy to a wide variety of targeted therapies. Advances in molecular genetics, and immunology, along with improved laboratory techniques, have led to the discovery of unique targets integral to the growth and proliferation of malignant cells. These revolutionary discoveries provide a foundation for the development of a new generation of anti-tumor agents. They include such new targeted, non-cytotoxic anticancer agents, as small-protein kinase inhibitors. Examples are the FDA approved tyrosine kinase inhibitors, Gleevec [Gleevec (imatinib mesylate); Novartis Pharmaceuticals Corp.], Iressa (gefitinib); Astra Zeneca Pharmaceuticals Inc.], and others that are in various stages of clinical development. [6]

Another example, with a different targeting mechanism, is the humanized monoclonal antibody bevacizumab (Avastin; Genentech). This agent targets and inhibits the function of the vascular endothelial growth factor (VEGF) that stimulates new blood vessel formation; and is therefore an anti-angiogenesis agent. It is currently approved as a first-line treatment for patients with metastatic colorectal cancer [7].

In addition to treatment of cancer there are new targeting agents known as biological response modifiers (BRMs). These target specific cytokines such as TNF-α and IL-1. These are being used for the treatment of diseases having an inflammatory component such as inflammatory bowel disease (IBD) and rheumatoid arthritis (RA). In the case of IBD they are the drugs of choice and for RA they are proving to be more effective than traditional disease-modifying antirheumatic drugs (DMARDs), especially when used in combination treatment such as with methotrexate. Four targeted drug therapies are approved for use in IBD and/or RA; three target TNF-α (1) etanercept; Enbrel, Amgen Inc. (A TNF-α decoy receptor). (2) infliximab; Remicade, Centocor Inc. and (3) adalimumab; Humira, Abbott Laboratories Inc. (both TNF-α monoclonal antibodies) and one that targets IL-1 (4) anakinra; Kineret, Amgen Inc. A reason why this problem is arising for the above three strong TNF-α inhibitors may be because TNF-α has a dual nature. It triggers the JNK-dependent pathway required for TNF-α-induced apoptosis and it also activates the protein. PI3-kinase, associated with cell survival that can block this very pathway. This dual nature thus sets up a "delicate life-death balance" in the cell. This finding originally brought with it increased hope for the use of TNF-α as a possible treatment against cancer. [8-10].

Strategies for targeting a single genes or proteins ignore a very important fact that the most, if not all of diseases involve a sophisticated network "system" [11]. For example, chemokines, a family of immune molecules related to IL-8 contains approximately 50 ligands and 20 receptors, often acting with redundancy, thus making selection of appropriate specific antagonists not only difficult, but lacking in long-term efficacy [12]. This argument is supported by the fact that many agents recently developed by targeting a specific molecule for the treatment of IBD are proving to be either insufficiently effective or totally ineffective. The examples of insufficient efficacy include p55-TNF binding protein, interferon α, β-a, interferon γ antibody, IL-12 antibody, P65 antisense oligo, G-CSF, GM-CSF, EGF, hGH, keratinocyte GF-2, CD4 antibody and α4β7-intergrin antibody whereas ineffectiveness includes IL-10, IL-11, ICAM-1 antisense, TNFR2 fusion protein Enbrel.

Many of single-targeted drugs mentioned above have been clinically proven effective in short term. For example, the treatment with biologic DMARDs relieves symptoms, inhibits the progression of structural damage, and improves physical function in patients with moderate to severe active RA. The 3 marketed TNF-α blocking agents have similar efficacy when combined with MTX, a widely used DMARD, in the treatment of patients with RA [13]. While providing significant efficacy and a good overall safety profile in the short and medium term in many patients with RA, these biologic treatments, however, may create serious problems and long-term side effects, such as on the liver, and still need to be evaluated. There has been a disturbing association between the use of both of Enbrel or Remicade and the development of lymphoma [14]. As described above, several reports have shown that patients treated with Enbrel or Remicade worsen their congestive heart failure and develop serious infection and sepsis, and increase exacerbations of multiple sclerosis and other central nervous system problems [15,16]. It is because many pathogenetic targets also have their multiple physiological functions and so can influence multiple cellular pathways simultaneously.

Nevertheless short-term side effects of all of the above drug treatments have been thought to be generally manageable. However, as they are relatively new agents, extended follow-ups are revealing unanticipated longer term results. As one example, imatinib, the first major drug in its class of specific inhibitors of tyrosine kinase receptors, has been found to be far less effective in patients who relapsed with accelerated and blast phases of CML [1]. It is therefore being recommended for use either as an alternative, or as an adjunct to donor lymphocyte infusions for patients with stable phase myeloid leukemia who relapse after allogeneic stem cell transplantation. It is not surprising that after a particular protein is targeted, resistance to a drug can evolve when cancer cells create a by-pass to the targeted activity. As a result, the emergence of resistance to imatinib has been recognized as a major problem in the treatment of CML. Therefore, regimens that combine imatinib with conventional chemotherapeutic agents, or with inhibitors of other signal transduction proteins that may be preferentially activated in CML cells are being pursued [17,18].

Two other very recent examples (2005) of drugs having a high degree of selectivity but nevertheless failed to live up to expectations or had unanticipated adverse events, are Iressa, a specific epidermal growth factor inhibitor which has a variety of side effects originally thought to be acceptable in light of its anti-cancer activity. However, Iressa failed to significantly prolong survival in comparison to placebo or in patients with adenocarcinoma [19]. The other, Tysabri, is a laboratory-produced monoclonal antibody which is the first of a new class of agents known as selective adhesion molecule (SAM) inhibitors. Tysabri was pulled from the market, and clinical trials of Crohn's diseases and rheumatoid arthritis were suspended because of two cases of progressive multifocal leukoencephalopathy (PML) [20].

An interesting and to the point of this paper, is the publication by Roth et al., in "Nature Reviews". The authors discuss the concept of using selective versus non-selective drugs for central nervous system (CNS) disorders. Since in most cases, multiple molecular lesions or signaling pathways are involved in pathogenesis of CNS disorders, the authors conclude that attempts to develop more effective treatments for diseases such as schizophrenia and depression by discovering drugs selective for single molecular target that is, "magic bullets" have, not surprisingly, been largely unsuccessful. They propose that "designing selectively non-selective drugs (that is, 'magic shotguns') that interact with several molecular targets will lead to new and more effective medications for a variety of central nervous system disorders" [21].

At the 10^th ^anniversary conference of The Society for Biomolecular Screening a presentation on single vs. combination drugs concluded that "During the last decade, the industry has followed an assumption that a single drug hitting a single target was the 'rational' way to design drugs. Now post-genomics biology is teaching us the fundamental limitations of the single target philosophy. Ironically many drugs on the market, discovered in 'black box' phenotype screens, are observed to bind potently to multiple targets and more so, this poly-pharmacology is key to their effect (personal communication; Andrew Hopkins, Pfizer Global Research & Development -United Kingdom; The Society for Biomolecular Screening, 10^th ^anniversary conference, September 14, 2004, Point/ Counterpoint – Polypharmacology: Single vs. Combination Drug)

A case in point is found in another recent study that combined an anti-EGFR monoclonal antibody and tyrosine kinase inhibitor, which target extra-cellular and intracellular domains of the receptor, respectively. Specifically, the combination of cetuximab (Erbitux, ImClone Systems, with either gefitinib (Iressa, AstraZeneca, or erlotinib (Tarceva, Genentech) across a variety of human cancer cells. The combination of cetuximab plus gefitinib or erlotinib enhanced growth inhibition over that observed with either agent alone. The study concludes that "together, these data suggest that combined treatment with distinct EGFR inhibitory agents can augment the potency of EGFR signaling inhibition. This approach suggests potential new strategies to maximize effective target inhibition, which may improve the therapeutic ratio for anti-EGFR-targeted therapies in developing clinical trials" [22].

At the American Society of Clinical Oncology annual meeting held in June, 2004, scientists discussed the concept of drug promiscuity. The focus here was in some cases, a drug that attacks multiple but limited targets may keep cancer cells from developing resistance [23].

The examination of the role of deregulated cell cycle progression and uncontrolled cellular proliferation can also elucidate the advisability of hitting multiple targets simultaneously in order to show superior efficacy. Uncontrolled proliferation is a condition found in a variety of diseased cells. It is a feature of cellular transformation, accompanied by the deregulation of Cyclin dependent Kinases (Cdks) involved in the control of the cell cycle, check points, and apoptosis which has a crucial role in the growth of both normal and malignant cells [24,25]. Apoptosis is an energy-dependent, normal process of cell death, based on morphological and biochemical changes in the cell that occurs in many biological conditions, but without concurrent pathological necrosis and inflammation [26].

Since immune cells in bone marrow (B-cells) or in the thymus (T-cells) undergo repeated cycling as part of their development, cell proliferation is a fundamental processwithin the immune system function [27-29]. Immune based diseases with a component of inflammation are risk factors for the development of several diseases including cancer, and it is known that anti-inflammatory agents markedly inhibit the development of cancer in humans; e.g., colon cancer [30]. Although the specific intracellular pathways and networks involved in these processes are not completely understood, at the molecular level, this is thought to be based on the combination of cellular transformation and cellular proliferation. The normal cell cycle and its intricate mechanism when deregulated (by germ or somatic factors) can lead to uncontrolled cellular proliferation, defective apoptosis, autoimmunity and inflammation and uncontrolled proliferation and defective apoptosis, can be viewed as both cause and consequence ofinflammation, cancer, and autoimmunity [31-34].

Multiple target drug screening approaches are being developed. As indicated, the pathogenesis of a disease is usually multi-factorial involving numerous risk factors and defective genes or proteins or signaling pathways out of balance with each other. There may be one major or most easily definable defective target (or pathway)externalized or typifiedfor a given disease, but collateral proteins which can act in a network rather than single pathway is likely to be involved and may lead to the emergence of backup (or redundant) systems to sustain the disease, or cause undesirable side effects [35-37]. This is a fundamental defect in the single target reasoning for therapeutic development.

To overcome this problem, a network approach has been described [20,38,39]. In this network model of pharmacological actions elements of the network represents various targets (proteins, RNA-s or DNA sequences), and each link corresponds to an interaction between proteins of the cell. Interestingly, application of this model revealed that multi-target drugs affect their targets only partially, which corresponds well with the presumed low-affinity interactions of these drugs with several of their targets. Low-affinity, multi-target drugs might have another advantage – weak links stabilize the systems buffering the changes after system-perturbations [39,40], thus lower side effects. Therefore, multi-target drugs and the network approach might become a useful mean of novel drug discovery.

Many successful drugs are promiscuous. The best known is aspirin or acetylsalicylic acid known to target any area where inflammation is present. In recent years, aspirin has surpassed the area of pain relief to also include activity as blood thinner, to reduce platelet aggregation in the prevention of cardiovascular disease, prevention of preeclampsia (an hypertensive disorders in pregnancy), and prevention of cancer. Aspirin's antiarthritic effect requires chronic or long-term therapy for pain and/or inflammation, e.g., rheumatoid arthritis, juvenile rheumatoid arthritis, systemic lupus erythematosus, osteoarthritis, ankylosing spondylitis, psoriatic arthritis, Reiter's syndrome, and fibrositis. Aspirin's major mode of activity is associated with its cyclooxygenase (COX) inhibitory activity. Cyclooxygenase enzymes are required for the conversion of arachidonic acid to prostaglandins. COX-2 mediates the inflammatory effects, and is induced by a wide spectrum of growth factors and proinflammatory cytokines. It is over-expressed in numerous pre-malignant and malignant lesions, including colorectal and prostate cancer. Recent papers suggest aspirin and salicylate at therapeutic concentrations inhibit COX-2 protein expression through interference with binding of CCAAT/enhancer binding protein beta (C/EBPbeta) to its cognate site on COX-2 promoter/enhancer. COX-2, is not normally produced in most tissues but is induced by a wide spectrum of growth factors and pro-inflammatory cytokines such as IL-1 and TNF-α, observed in such cell types as synoviocytes, endothelial cells, chondrocytes, osteoblasts, and monocytes/macrophages. Expression of genes, such as inducible nitric oxide synthase and interleukin-4, may be inhibited by aspirin and salicylate by a C/EBP-dependent mechanism Aspirin at supra-pharmacological concentrations inhibits NF-kappaB-mediated gene transcription and protects tissue from injury. Other pathways yielding other effects are likely but as yet, uncovered [41-46].

## Summary

As discussed, although the basic mechanistic activity of a drug in a given cell may be relatively simple, the resultantactivity it produces in the human body can be highly complex. An activity can involve the balance and interplay of multiple signaling networks and result in unintended consequences. The pathogenesis of a disease is usually multi-factorial involving numerous risk factors and defective proteins or proteins out of balance with each other. There may be one major or most easily definable defective target (or pathway)externalized or typifiedfor a given disease, but collateral proteins which can act in a network rather than single pathway are likely to be involved and may lead to the emergence of backup (or redundant) systems to sustain the disease, or cause undesirable side effects [35-37]. This is a fundamental defect in the single target reasoning for therapeutic development.

Consequently, it is conceivable that a perfect drug is not one that has selectivity for one protein, nor one molecular mechanism. Such super-selectivity gives a drug maximal efficacy and minimal adverse effects or toxicity only if the target of drug is the only one involved in the pathogenesis of a disease or the target is presented only in the targeted tissues. This, situation apparently occurs only rarely. Perhaps, therefore, an ideal drug may be one whose efficacy is based not on the inhibition of a single target, but rather on the rebalancing of the several proteins, or events, that contribute to the etiology, pathogeneses, and progression of diseases, i.e., in effect a promiscuous drug

Based on the above discussion, one may infer that for control of some diseases, an "ideal" drug could very well be one that can hit more than one target, and stimulate/inhibit more than one molecular activity but do so at a concentration sufficiently low not to induce undesirable side effects. When a disease is based on an imbalance of several proteins or is genetically, physiologically, and ultimately pharmacologically heterogeneous, the logic for promiscuity in drugs is intuitive. However, it remains to be seen if drug promiscuity will be superior to targeted drugs when a disease may be homogeneous. The ability to detect true homogeneity is the crux of this dilemma.

Nevertheless, when considering all the ramifications of a rare, ideal case of a single agent affecting a single molecular-driver, andeven then, having concomitant serious side effects, one wonders if a broad based and very expensive screening process used to search for truly selective drugs is a beguiling, but distracting and perhaps a deluding, application of large amounts of research money

Briefly, a promiscuous drug may be advantageous because:

• Different diseases may have related etiology or similar pathological alteration.

• Multiple factors contribute to the pathogenesis of a particular disease.

• Redundancy widely exists in biologically critical pathways

• Being promiscuous is not necessarily more toxic.

• Promiscuous drugs do not necessarily completely shut down or excessively activate a pathway or network

Such drugs are the result of the emerging use of Network Biology. Network Biology is based on the understanding of how cellular molecules and their interactions determine the function of complex cellular machinery, both by themselves in isolation, as well as with other nearby cells. Various types of molecular interaction webs (including protein-protein interaction, metabolic, signaling and transcription-regulatory networks) emerge from the sum of these interactions that together are principal determinants of the system-scale behavior of the cell [47].

Further, new technology is being developed related to screening for the discovery of promiscuous drugs. An example is the new cell-based high-throughput technology for screening chemical libraries against several potential cancer target genes in parallel. This multiplex gene expression (MGE) analysis provides direct and quantitative measurement of multiple endogenous mRNAs using a multiplexed detection system coupled to reverse transcription-PCR [48].

Several brief examples of promiscuous drugs currently under development include:

### 1). SU11248 (Pfizer)

SU11248 is a small protein receptor tyrosine kinase inhibitor that interferes with several cellular signaling pathways. It has with direct anti-tumor as well as antiangiogenic activity via its multi-targeting process; the vascular endothelial growth factor (VEGF), platelet-derived growth factor (PDGF), KIT, and FLT3 receptor tyrosine kinases.

### 2). Bay 43-9006 (Bayer)

BAY 43-9006 inhibits a variety of kinase receptors, including VEGF and PDGF. It is the first agent to target both the RAF/MEK/ERK signaling pathway to inhibit cell proliferation and the VEGFR-2/PDGFR-β signaling cascade to inhibit tumor angiogenesis.

### 3). NTI-2001 (Natrogen Therapeutics)

NTI-2001 regulates two very different avenues associated with disease pathogeneses. It modulates several cytokines, e.g., TNF-α only moderately and so should not do so to the degree of being a cancer causative, but its modulation is effective for restoring the cytokine balance, key for the treatment of inflammatory based diseases. In addition to regulating such cytokines as Il-1β, Il-6, and Il-10 it can inhibit Cdks involved in cellular transformation and cellular proliferation. The restoration of cytokine balance and Cdks inhibition, are both important factors not only in cancer but in the pathogenesis of other inflammatory diseases.

## Competing interests

The authors have a financial interest in Natrogen Therapeutics Corporation that is developing the drug described in the last paragraph of this paper. The company is a small, private organization however it is conceivable that in the future, they can gain or lose financially from the publication of this manuscript.

## Authors' contributions

Both authors of the paper, SK Mencher and LG Wang have contributed equally to the paper.
